# Dissecting the Sequential Assembly and Localization of Intraflagellar Transport Particle Complex B in *Chlamydomonas*

**DOI:** 10.1371/journal.pone.0043118

**Published:** 2012-08-10

**Authors:** Elizabeth A. Richey, Hongmin Qin

**Affiliations:** Department of Biology, Texas A&M University, College Station, Texas, United States of America; Purdue University, United States of America

## Abstract

Intraflagellar transport (IFT), the key mechanism for ciliogenesis, involves large protein particles moving bi-directionally along the entire ciliary length. IFT particles contain two large protein complexes, A and B, which are constructed with proteins in a core and several peripheral proteins. Prior studies have shown that in *Chlamydomonas reinhardtii*, IFT46, IFT52, and IFT88 directly interact with each other and are in a subcomplex of the IFT B core. However, *ift46*, *bld1*, and *ift88* mutants differ in phenotype as *ift46* mutants are able to form short flagella, while the other two lack flagella completely. In this study, we investigated the functional differences of these individual IFT proteins contributing to complex B assembly, stability, and basal body localization. We found that complex B is completely disrupted in *bld1* mutant, indicating an essential role of IFT52 for complex B core assembly. *Ift46* mutant cells are capable of assembling a relatively intact complex B, but such complex is highly unstable and prone to degradation. In contrast, in *ift88* mutant cells the complex B core still assembles and remains stable, but the peripheral proteins no longer attach to the B core. Moreover, in *ift88* mutant cells, while complex A and the anterograde IFT motor FLA10 are localized normally to the transition fibers, complex B proteins instead are accumulated at the proximal ends of the basal bodies. In addition, in *bld2* mutant, the IFT complex B proteins still localize to the proximal ends of defective centrioles which completely lack transition fibers. Taken together, these results revealed a step-wise assembly process for complex B, and showed that the complex first localizes to the proximal end of the centrioles and then translocates onto the transition fibers via an IFT88-dependent mechanism.

## Introduction

Cilia and flagella are hair-like organelles extending from the basal bodies of almost all eukaryotic cells. These cellular appendages play important roles for cellular homeostasis and development. Failure to build or maintain functioning cilia leads to a wide spectrum of human diseases and disorders collectively known as ciliopathies, which include hydrocephalus, polycystic kidney disease, primary ciliary dyskinesia, Bardet Biedl Syndrome, etc [1], [2].

The assembly and function of the flagella relies on intraflagellar transport (IFT), the conserved bi-directional movement of linear trains of IFT particles along the axoneme [3]. The IFT particle is composed of two complexes, A and B, which play overlapping yet distinct functions for the traffic of IFT. Complex B is essential for the anterograde IFT, while complex A appears to mediate the retrograde movement [4], [5], [6], [7], [8]. There are at least 6 subunits in complex A and 13 in complex B [9], [10]. In *Chlamydomonas*, both IFT A and B complexes contain a core complex and several peripheral proteins. The complex A core refers to the smaller complex found in a complex A mutant *ift121* [11]. The complex B core is an 11 S complex that stays intact in high ionic conditions [12].

Using the *E. coli* co-expression system, previous studies have revealed that the complex B core contains at least two tetrameric subcomplexes IFT81/74/27/25 and IFT88/70/52/46 [13]. IFT52 was suggested to bridge the two tetramers since it directly interacts with IFT81/74/27/25 [13]. IFT27/25 and IFT81/74 form two stable dimer complexes [12], [14], [15]. The IFT88/70/52/46 tetramer contains many strong interactions. IFT52, IFT46, and IFT88 interact with each other [13], [16], while IFT52 and IFT46 also interact with IFT70 [13], [17]. Pair-wise interaction assays have provided some detailed information as to the structure of the complex B core. However, little is known as to how the interactions among the subcomplexes contribute to the *in vivo* assembly of the functional complex B.

To date, many IFT protein mutants have been identified in various organisms. These mutants all have defects in ciliogenesis. However, the severity of such defects varies among the IFT protein mutants. For example, in *Caenorhabditis elegans*, the cilia of *osm-6/ift52* and *osm-5/ift88* mutants are highly stunted with no ciliary axonemal microtubules beyond the transition zone [18]. In contrast, *dyf-6/ift46* [19], *dyf-1/ift70* [20], *ift74*, and *ift81* [21], [22] mutants are capable of assembling the middle segment of the chemosensory amphid and phasmid cilia, and IFT motility is intact in the remaining cilia. So far, the function of IFT88 has been studied in many different species. The IFT88 mutants of *Chlamydomonas* [23], *Drosophila* [24], and mouse [25] are all incapable of assembling cilia, demonstrating that IFT88 plays an essential and conserved role in ciliogenesis. The *Chlamydomonas* mutant of IFT52, *bld1*, is completely flagella-less [26], which is consistent with the phenotype seen in *C. elegans*. The *Chlamydomonas ift46* mutant [27] and the *Tetrahymena dyf-1/ift70* [28] mutant have very short flagella with severely disrupted axonemal structures. The fact that neither *dyf-6/ift46* nor *dyf-1/ift70* are completely flagella-less indicates IFT46 and IFT70 do not completely abrogate IFT and ciliogenesis.

*Chlamydomonas* IFT46, IFT52 and IFT88 are in the same subcomplex of the IFT B core and can form a stable trimetric complex [16]. Multiple interactions have been found among these three subunits, thusit was expected that deletion of any of the components would destroy the entire complex, and stop IFT. However, their mutants differ in phenotype as *ift46* mutants are able to form short flagella [27], while the other two mutants lack flagella completely [23], [26]. This led us to ask, what are the different roles that IFT46, IFT52, and IFT88 play in IFT, and how can these differences lead to presence or absence of flagella in these mutants? By analyzing the *Chlamydomonas* mutants, we conclude that *ift46* is able to form short flagella because it still forms a functional, yet unstable, complex B. Although *ift88* maintains IFT protein stabilities and forms the complex B core, its inability to localize the complex B to the initiation site for ciliogenesis may account for its failure to assemble flagella. The *bld1* mutant has decreased IFT stabilities, a disrupted complex B core, and a failure of localizing complex B proteins to the transition fibers of the basal bodies, all of these factors leading to a flagella-less phenotype. These results provide insight into the *in vivo* assembly process of complex B and reveal for the first time that IFT complex B first localizes to the proximal end of the centrioles and then translocates onto the transition fibers via an IFT88-dependent mechanism.

## Results

### IFT46 is not Essential for Complex B Assembly

**Figure 1:**
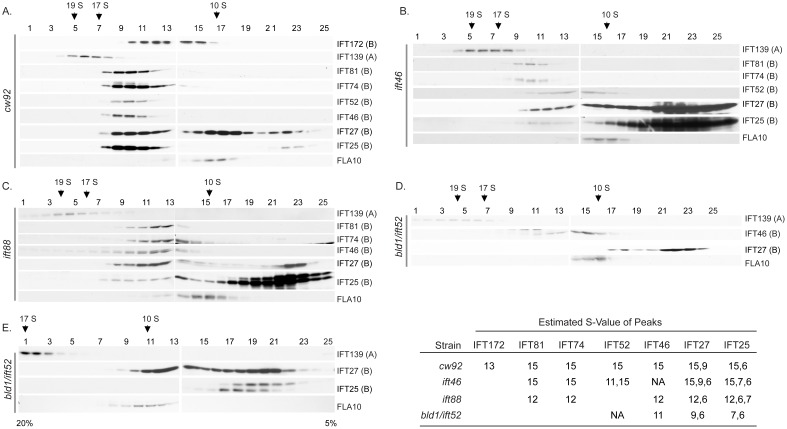
Complex B assembly in *ift46, bld1*, and *ift88* mutant cells. 10–25% sucrose gradients were used for all strains, along with one 5–20% gradient for *bld1*. (A) *cw92*, a cell wall defective mutant which has wild type IFT particles, was used as the control. IFT complex B sedimented at about 15 S. IFT27 had additional peaks at 9 S and 6 S. IFT25 had an additional peak at 6 S. IFT172 peaked at about 13 S. (B) *ift46* mutant still assembled a nearly complete complex B. IFT52 had an additional peak at 11 S. Also, a high concentration of IFT25 and IFT27 was found to peak at S-values as low as 6 S. There was an additional peak for IFT27 at about 9 S. (C) *ift88* mutant had a 12 S complex. Also note that like *ift46*, this strain also had a large amount of IFT25 and IFT27 that peaked as low as 6 S, however lacked the 9 S peak for IFT27. (D, E) In *bld1* mutant, IFT46 peaked at approximately 11 S and IFT25 and IFT27 peaked at 6 S. IFT27 had an additional peak at 9 S. No other B proteins were detected due to low amounts of proteins.

Sucrose density gradients were used to investigate the assembly status of IFT complex B in *ift46, ift88*, and *bld1/ift52* mutants (**Fig. 1**). In wild type (**Fig. 1A**), all probed complex B core proteins cosedimented together at about 15 S, representing the preassembled complex B. Consistent with previous findings [7], [9], [29], IFT172, a peripheral B protein, peaked at about 13 S, confirming that this peripheral protein can readily dissociate from complex B without disrupting the structure of complex B.

IFT B proteins from *ift46* mutant showed a very similar sedimentation pattern to wild type (**Fig. 1B**). Like wild type, all the IFT B proteins peaked at about 15 S. However, IFT52 had an additional peak at about 11 S, indicating that this protein exists in two separate complex forms. The 11 S complex likely serves as an intermediate transition complex, while the 15 S complex is a completely or almost completely assembled complex B. IFT74 and IFT81, however, were only found at the 15 S peak together with IFT52 and there were no free IFT74 and IFT81 proteins. There was a dramatic increase in the amount of 6 S IFT25/27, indicating that a significant fraction of IFT25/27 freely exists. Based on these results, we concluded that IFT46 is not required for assembly of the IFT complex B. Since short flagella do form in *ift46* mutant [27], the 15 S complex B is likely functional and capable to support flagellar assembly.

When co-expressed in *E. coli*, complex B proteins assemble into several subcomplexes including two tetrameric subcomplexes, IFT81/74/27/25 and IFT88/70/52/46, and two dimer complexes, IFT27/25 and IFT81/74 [12], [13], [14], [15]. The *ift46* mutant contained the 15 S complex B, an 11 S transitional complex, and a free IFT27/25 complex (**Fig. 1B**). IFT52, IFT25, and IFT27 were found in the 11 S complex, thus the 11 S complex likely is comprised of IFT88/70/52/27/25. Interestingly, IFT81 and IFT74 were only detected in the 15 S complex, with no free IFT81/74 complex. Therefore, unlike IFT27/25 subcomplex, the IFT81/74 complex does not exist independently. This result is consistent with previous findings that the full length IFT81/74 expressed in *E. coli* is insoluble [13]. The IFT81/74 complex may not be stable without being bound to the complex B core proteins and thus quickly degrades *in vivo*. Taken together, these results showed that the complex B core appears to assemble in a sequential order: the IFT88/70/52/46/27/25 subcomplex would have to form first in order to allow IFT81/74 to assemble onto the core.

In the *ift46* mutant, complex B proteins still assembled into the 15 S complex, which clearly showed that IFT46 is not required for assembly of the IFT complex B. However, without IFT46 the efficiency for assembly is reduced, which allows the accumulation of the 11 S intermediate transitional complex (**Fig. 1B**). IFT46 is likely assisting in the efficient binding of the subcomplexes IFT81/74 and IFT88/70/52/46/27/25 to form the complex B core.

### IFT88 is not Essential for Complex B Core Assembly, but is Required for Peripheral Proteins to Attach to the B Core

In *ift88* mutant cells, complex B proteins formed a small complex sedimenting at 12 S fractions (**Fig. 1C**) [30], indicating the complex B core is still intact, but at least some peripheral proteins fail to assemble onto the core. Therefore, IFT88 is not required for IFT complex B core assembly, but rather bridges peripheral proteins to the core.

### IFT52 is Essential for Complex B Core Assembly

**Figure 2:**
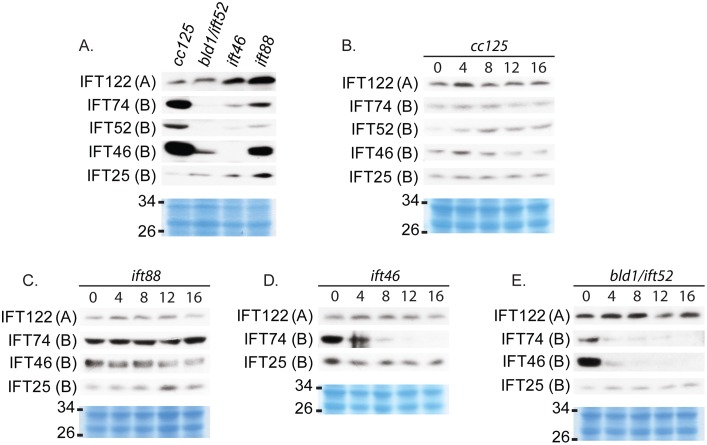
IFT46 and IFT52 are important for IFT B protein stability, while IFT88 is not. Western blot analysis of whole cell extracts of untreated strains or strains treated with cycloheximide. Coomassie-stained gels (in blue color) were used to show equal protein loadings. (A) *Bld1*, *ift46*, and *ift88* have different levels of reduction of IFT B proteins, and an increase or no change in IFT A proteins; *Bld1* shows the most extreme change, while *ift88* shows the least extreme change compared to wildtlype. (B–E) Cells were treated with cycloheximide, a protein synthesis inhibitor, to measure degradation rates of existing proteins within 16 hours of treatment. (B) In wild type (*cc125*) IFT A and IFT B proteins remain stable throughout the time course. (C) *Ift88* has near wild type stabilities of IFT A and IFT B proteins. (D) *Ift46* has stabilities much like *bld1* for IFT A and IFT B proteins. (E) *Bld1* has an extreme decrease in IFT B protein stabilities, with the exception of IFT25, and no change in stability of IFT A proteins.

Previous pair-wise interaction studies have shown that IFT52 binds directly to the IFT81/74/27/25 complex [13], which leads to the possibility that IFT52 serves as a bridge to connect the two tetrameric complexes, IFT88/70/52/46 and IFT81/74/27/25, to form the complex B core. However, it is unclear whether association of these two tetramers solely depends on IFT52. Unlike the *ift46* and *ift88* mutants, the *bld1* cells showed a much disrupted complex B (**Fig. 1D, E**). Since this mutant had extremely reduced amounts of IFT proteins (**Fig. 2A**), we were only able to detect IFT46, IFT25, and IFT27 on the gradients. IFT46 peaked at about 11 S. This 11 S complex likely contained IFT88, IFT70, and IFT46. Interestingly, unlike the 11 S intermediate transition complex found in the *ift46* mutant (**Fig. 1B**), IFT25 and IFT27 were not detected in the 11 S fractions in the *bld1* mutant (**Fig. 1D, E**). Therefore, the 11 S complex of *bld1* mutant was only composed of the IFT88/70/46 subcomplex, but not IFT27/25. The intermediate transition complexes, IFT27/25 and IFT88/70/46, stayed as two separated complexes that failed to assemble into the complex B core in the *bld1* mutant. Based on the results from the *ift46* and *bld1* mutant gradients, we concluded that IFT52, but not IFT46, is essential for the binding of IFT88/70/52/46 and IFT27/25. During complex B core assembly, IFT88/70/52/46 and IFT27/25 must assemble into the complex IFT88/70/52/46/27/25 to allow the subsequent binding of IFT81/74.

### The Sedimentation Pattern of IFT27 is Unique from other Proteins

Unexpectedly, IFT27 formed at least three peaks in wild type (**Fig. 1A**), which is different from what we have observed previously [14]. Prior studies show that IFT27 forms two peaks, one with complex B, and the other in the dimer complex IFT27/25. The third complex that we observed sedimented at approximately 9 S. The IFT B proteins that we probed did not cosediment at 9 S, thus this 9 S complex contained IFT27 bound to other unknown proteins. The 9 S complex was observed not only in wild type, but also in *ift46* and *bld1* mutant cells (**Fig. 1B, C and D**). However, the 9 S complex was not observed in *ift88*. One possible explanation to this phenomenon is that in wild type, *bld1*, and *ift46* mutant, IFT27 forms a subcomplex with one or a group of the peripheral proteins, whereas it is unable to do so in the *ift88* mutant since IFT88 may be required to recruit these peripheral proteins and allow binding to IFT27. Checking the sedimentation patterns of peripheral proteins to see if they peak with the 9 S IFT27 subcomplex will allow us to test this idea.

IFT25 is a highly phosphorylated protein. The higher molecular weight band represents the phosphorylated form since it migrates slower on the gel [14]. The phosphorylated and the non-phosphorylated IFT25 were readily separated on the *ift88* and *bld1* gradients (**Fig. 1C, D and E**). Interestingly, IFT27 cosedimented with the phosphorylated form of IFT25 at approximately 6 S, but not with the non-phosphorylated form. These results hinted that the phosphorylation of IFT25 regulates the binding between IFT27 and IFT25.

### IFT46 and IFT52 are Important for IFT Complex B Protein Stabilities, while IFT88 is not

Based on western blot analysis of whole cell protein extracts (**Fig. 2A**), the mutants *ift46*, *bld1*, and *ift88* all showed greatly reduced amounts of complex B proteins when compared to wild type cells. IFT25 was an exception and followed a similar pattern to IFT A rather than B proteins. This may be because IFT25/27 subcomplex is stable apart from the core complex and therefore if IFT B proteins are reduced, it is not affected as the other B proteins. Complex A proteins were not reduced but rather were either increased, as in *ift46* and *ift88*, or remained constant, as in *bld1*. This is consistent with previous findings that complex B mutants often have overexpressed complex A proteins and under-expressed complex B proteins [17], [23], [27], [31]. Of the three mutants, *bld1* had the greatest reduction in complex B proteins, suggesting that IFT52 is extremely important for maintaining cellular levels of complex B proteins. IFT46 and IFT88 are also very important for maintaining cellular levels, but to a lesser extent than IFT52.

A cycloheximide treatment time-course experiment was conducted to measure the degradation and turnover rates of IFT proteins in these mutants. IFT proteins were very stable in wild type cells with no noticeable degradation within 16 hours of treatment (**Fig. 2B**). The *ift88* mutant cells showed a slight decrease but near wild type stabilities of IFT proteins (**Fig. 2C**). In contrast, except IFT25 and IFT27 (data not shown), *ift46*, and *bld1* cells had a dramatic decrease in IFT B protein stabilities, with a dramatic drop in the amount of IFT B proteins within 4 hours of treatment (**Fig. 2D, E**). Thus IFT46 and IFT52 are important for preventing degradation of IFT complex B proteins.

### IFT Particle Proteins and the Anterograde Motor Fla10 have Independent Stabilities

**Figure 3:**
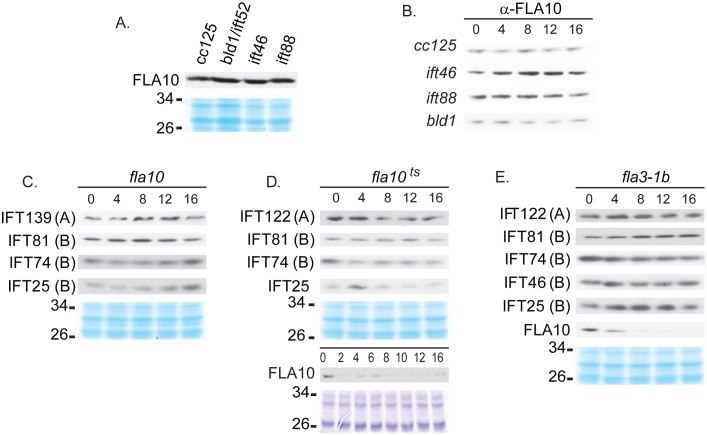
IFT protein and motor stabilities are independent from each other. Western blot analysis of whole cell extracts of untreated strains or strains treated with cycloheximide. Coomassie-stained gels (in blue color) were used to show equal protein loadings. (A) Untreated cells of *ift46, ift88,* and *bld1* had the same or slightly higher amount of FLA10 compared to wild type *cc125*. (B) The FLA10 protein was stable throughout 16 hrs of cycloheximide treatment in the wild type cells as well as in the mutants *ift46, ift88*, and *bld1*. (C–E) IFT A and B proteins were stable in *fla10* null (C), *fla10^ts^* (D), and *fla3-1b* (E) mutants throughout the cycloheximide treatment.

To understand whether the defective complex B affects the expression of the anterograde IFT motor Fla10, the whole-cell amount of FLA10, a subunit of the Fla10 motor, was checked in *ift46*, *bld1*, and *ift88* mutants. FLA10 appeared either at the same level or slightly higher than that of wild type (**Fig. 3A**). A cycloheximide time-course treatment showed that FLA10 was very stable in wild type cells, and in *ift46*, *bld1*, and *ift88* mutants (**Fig. 3B**). Thus, the reduced amounts of complex B proteins (**Fig. 2A**) or reduced stabilities of B proteins (**Fig. 2D, E**), does not affect the stability of FLA10.

A reciprocal cycloheximide time-course treatment experiment of *fla10*, a null mutant of a FLA10 allele, was carried out to examine if the lack of FLA10 affects the stabilities of complex A or B proteins. Throughout the 16 hour cycloheximide treatment there was no reduction in IFT proteins (**Fig. 3C**). Taken together, these results demonstrated that the stability of IFT particle proteins and the anterograde motor Fla10 are regulated through independent mechanisms.

The anterograde motor Fla10 is a heterodimer kinesin-II, containing two motor subunits FLA10 and FLA8, and an associated protein FLA3 [32]. *Fla10^ts^* and *fla3-1b* are two temperature sensitive (ts) mutants of the subunit FLA10 [33] and FLA3 [34], respectively. At the permissive temperature, the mutant proteins FLA10 and FLA3 are capable of supporting the flagella assembly and maintenance, but are inactivated at the non-permissive temperature, leading to the loss of flagella [9], [33], [34], [35]. We found that at the permissive temperature, when these two mutants were treated with cycloheximide, FLA10 greatly reduced in amount within four hours of the treatment in both mutants (**Fig. 3D, E**), indicating that the FLA10 protein is highly unstable and has a quick turnover rate. Since not only the *fla10^ts^*, but also the *fla3-1b* mutant, had reduced stabilities of FLA10, it is likely that each subunit contributes to the stability of the heterodimer Fla10-kinesin-II complex. Reduced stability of a single Fla10-kinesin-II complex protein causes higher turnover rate of the entire complex.

### IFT52 and IFT88 are Required for Localization onto the Basal Body

**Figure 4:**
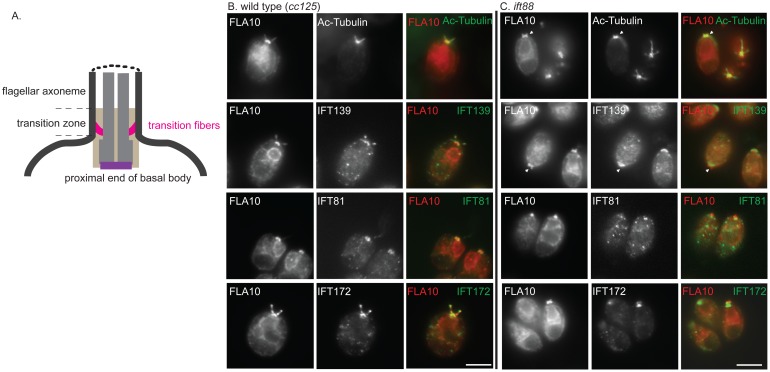
IFT88 is required for localization of complex B proteins to transition fibers of basal bodies. (A) A cartoon to illustrate the position of the transition fibers in the transition zone and the proximal end of a basal body. Immunofluorescent stains showing localization patterns of IFT proteins within the wild type cells (B) and the *ift88* mutant cells (C). The localization of FLA10 showed the cellular sites of transition fibers [36]. Acetylated tubulin (Ac-Tubulin) marked the basal bodies and the flagella emanating from the basal bodies (in wild type). (B) Both A (IFT139) and B (IFT81 and IFT172) proteins colocalized with FLA10 at the basal bodies and extended into the flagella in wild type cells. (B) In *ift88*, IFT139, an IFT A protein, colocalized with FLA10 at the transition fibers. FLA10 was also detected in the very distal part of the transition zone (arrows). Two B proteins, IFT172 and IFT81, however, localized only to the proximal ends of basal bodies. Scale bar, 10 µm.

**Figure 5:**
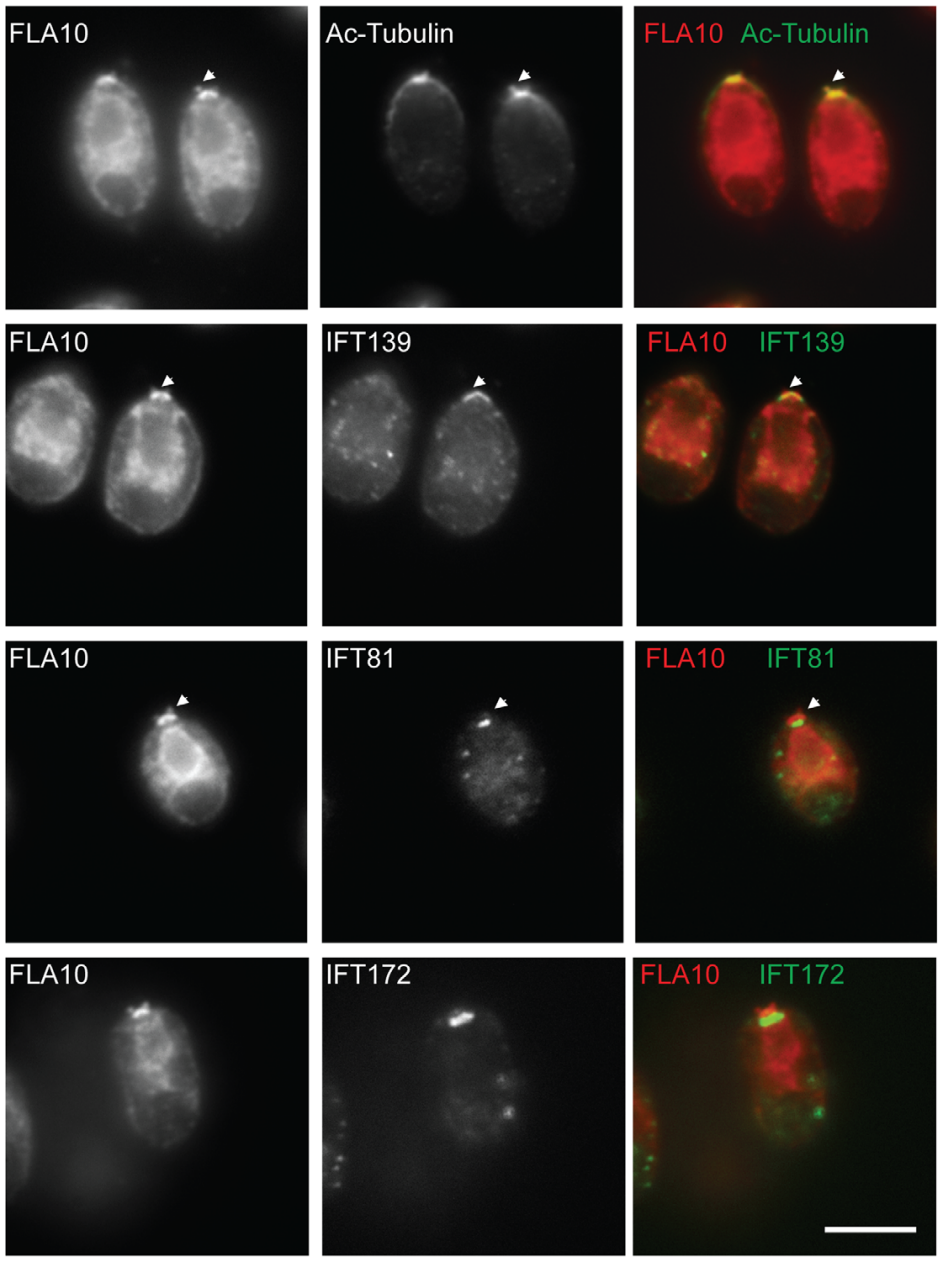
Complex B proteins localize to the proximal end of the basal bodies. Immunofluorescent stains showing localization patterns of IFT proteins within the *bld1* mutant. Acetylated tubulin (Ac-Tubulin) showed the cellular location of basal bodies. FLA10 and IFT139 correctly localized onto the transition fibers of the basal body. FLA10 was also found at the very distal part of the transition zone (arrows). IFT81 and IFT172, two complex B proteins, were found at the proximal end of the basal bodies, but not on the transition fibers. Scale bar, 10 µm.

It is perplexing that although the complex B core is intact, the *ift88* mutant is completely flagella-less. We reasoned that the failure to assemble flagella could be due to mislocalization of IFT particle proteins. In wild type cells, IFT particle proteins localize to transition fibers that extend from the distal portion of the basal body to the cell membrane [36] (**Fig. 4A**). The side view of the staining of IFT particle proteins and FLA10 is seen as a band beneath each flagellum ( [9], [27], [36] also see **Fig. 4B**). Similar to wild type, IFT139 (an IFT A protein) colocalized with FLA10 onto the basal bodies in the *ift88* mutant cells (**Fig. 4C**). Interestingly, in *ift88* mutant, FLA10 not only was seen as two bands at the apical end of the cell, but also appeared to extend further beyond the transition fibers and appeared to cover the entire transition zone (**Fig. 4C** arrows). In contrast, IFT139 was only observed as two bands at the transition fibers. The same localization patterns were also observed in *bld1* mutant (**Fig. 5** **,** arrows). These results indicated that the anterograde motor FLA10-kinesin-II could enter the flagellar compartment without attaching to IFT particles.

In wild type cells, IFT complex B proteins, IFT81 and IFT172, which were seen as two straight bands beneath the flagella, colocalized with FLA10 (**Fig. 4B**). However, in *ift88* mutant, both IFT81 and IFT172 no longer colocalized with FLA10, but were instead localized to the proximal ends of the basal bodies, and failed to localize onto the transition fibers (**Fig. 4C**). Thus, although the *ift88* mutant contained the intact complex B core (**Fig. 1C**), the failure to dock the IFT complex B onto transition fibers, the initiation site for flagellar assembly, could explain why the *ift88* mutant cells completely lack flagella [23].

To check if the proximal end basal body localization of complex B proteins requires them to be in the B core complex, the *bld1* mutant, which contains a disrupted complex B core (**Fig. 1D, E**), was used in immunofluorecent staining analysis (**Fig. 5**). Similar to *ift88* mutant, both IFT81 and IFT172 localized to the proximal ends of the basal bodies, but failed to dock onto the transition fibers (**Fig. 5**). Thus, it is likely that individual complex B proteins are able to localize to the proximal ends of basal bodies, but their subsequent translocation onto the transition fibers require them to be in a completely assembled complex B.

### The Basal Body Transition Fibers are not Involved in the Apical Localization of IFT Particles

**Figure 6:**
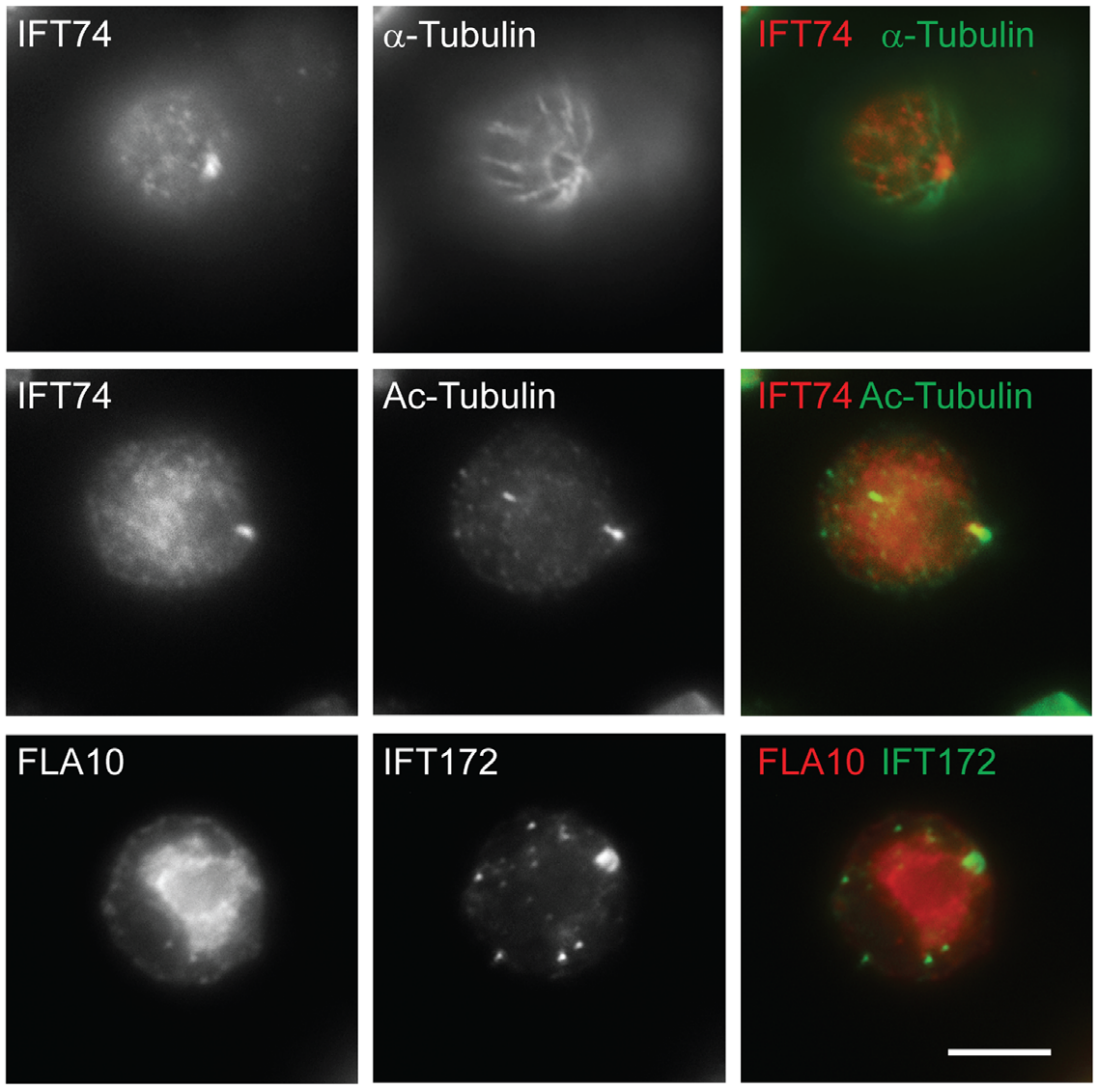
Transition fibers are not essential for the localization of IFT particles to the basal bodies. Both IFT74 and IFT172 localize to the defective centrioles in *bld2* mutant. FLA10, a subunit of the anterograde motor is no longer localized to the defective centrioles, which is consistent with the previous finding [9]. The staining of acetylated tubulin (Ac-Tubulin) labeled the rootlet microtubules and basal bodies, and α-tubulin showed all microtubules. Scale bar, 10 µm.

Both the *ift88* and *bld1* mutants have normal transition zone fibers [23], [26]. However, IFT complex B proteins no longer localize to the transition fibers, but rather to the proximal end of the basal body in these mutants (**Figs. 4** **and** **5**). Thus, we reasoned that the basal body transition fibers, although they serve as the initiation site for IFT movement, are not essential for targeting complex B proteins to the apical basal body region. The centrioles in *bld2* mutant lack the B- and C-tubule of the triplet microtubule blades. Consequently, the *bld2* centrioles do not have transition fibers and do not anchor to the apical membrane [37]. In *bld2* cells, IFT particle complex B proteins IFT172 and IFT74 still localized to the defective centrioles (**Fig. 6**). Thus, the transition fibers are not essential for the localization of IFT particles to the basal bodies.

### Complex B Protein IFT74 Localizes to the Rootlet Microtubules

**Figure 7:**
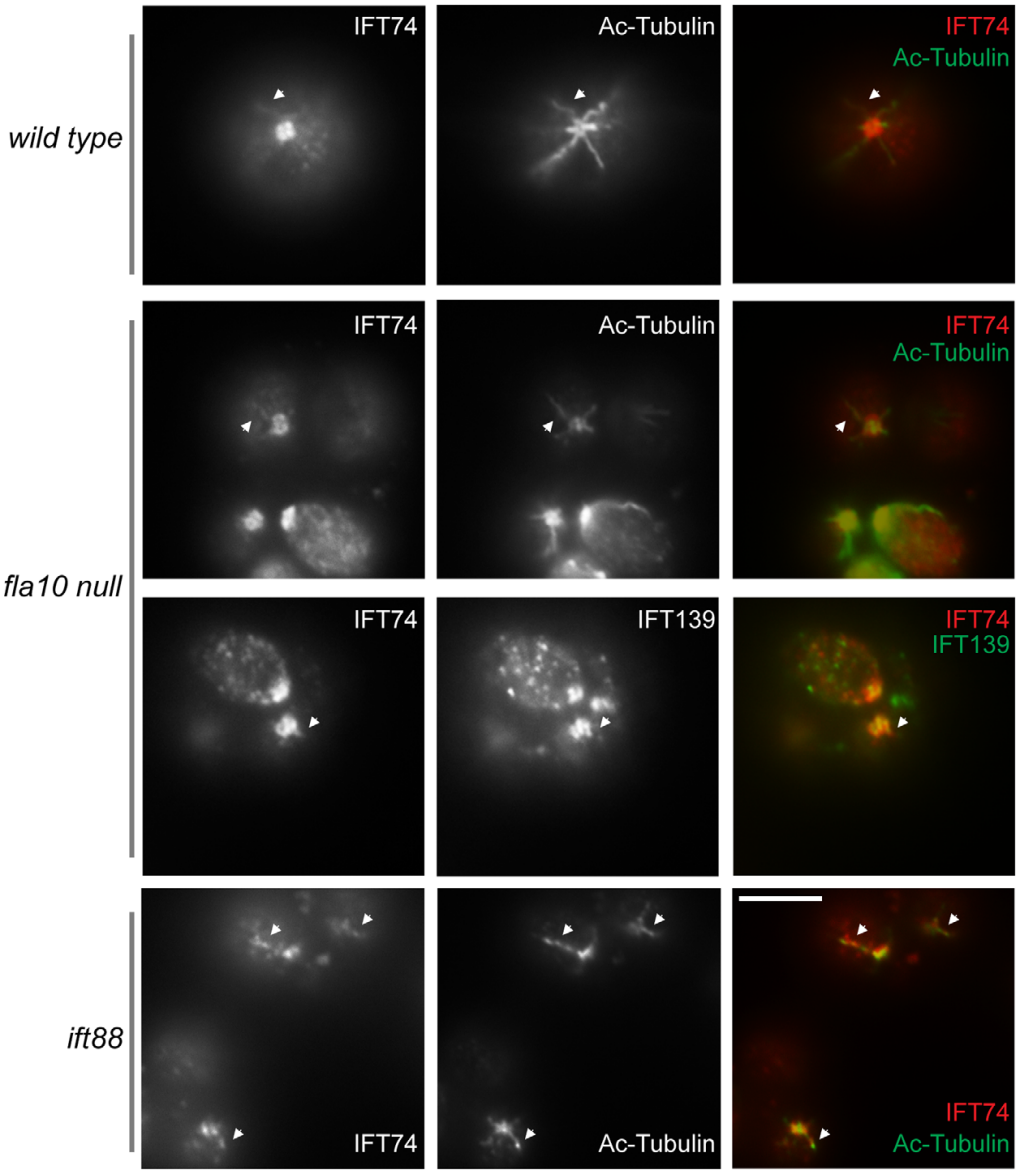
IFT74 co-localizes with rootlet microtubules. Immunofluorescent analysis showed IFT74 co-localized with rootlet microtubules in wild type, *fla10 null* and *ift88* cells. IFT74 and IFT139 also were found in dots throughout the cytoplasm. The staining of acetylated tubulin (Ac-Tubulin) marked the rootlet microtubules and basal bodies. The arrows point to the sites co-stained with both IFT74 and acetylated tubulin antibodies. Scale bar, 10 µm.

In *Chlamydomonas,* the stable rootlet microtubules and cytoplasmic microtubules have their minus ends focused on the proximal ends of basal bodies [37]. Co-immunofluorescent staining analysis found complex B protein IFT74 localized to the rootlet microtubules (**Fig. 7**). Moreover, IFT74 frequently was observed in dots throughout the cytoplasm (**Fig. 7**). Those dots appeared in lines, suggesting they attached to certain filaments. It is possible that these filaments are cytoplasmic microtubules. However, better resolution microscopic analysis and additional experiments are required to make such a conclusion.

## Discussion

### Assembly of IFT Complex B is Sequential and Basal Body Localization is a Two-step Process

**Figure 8:**
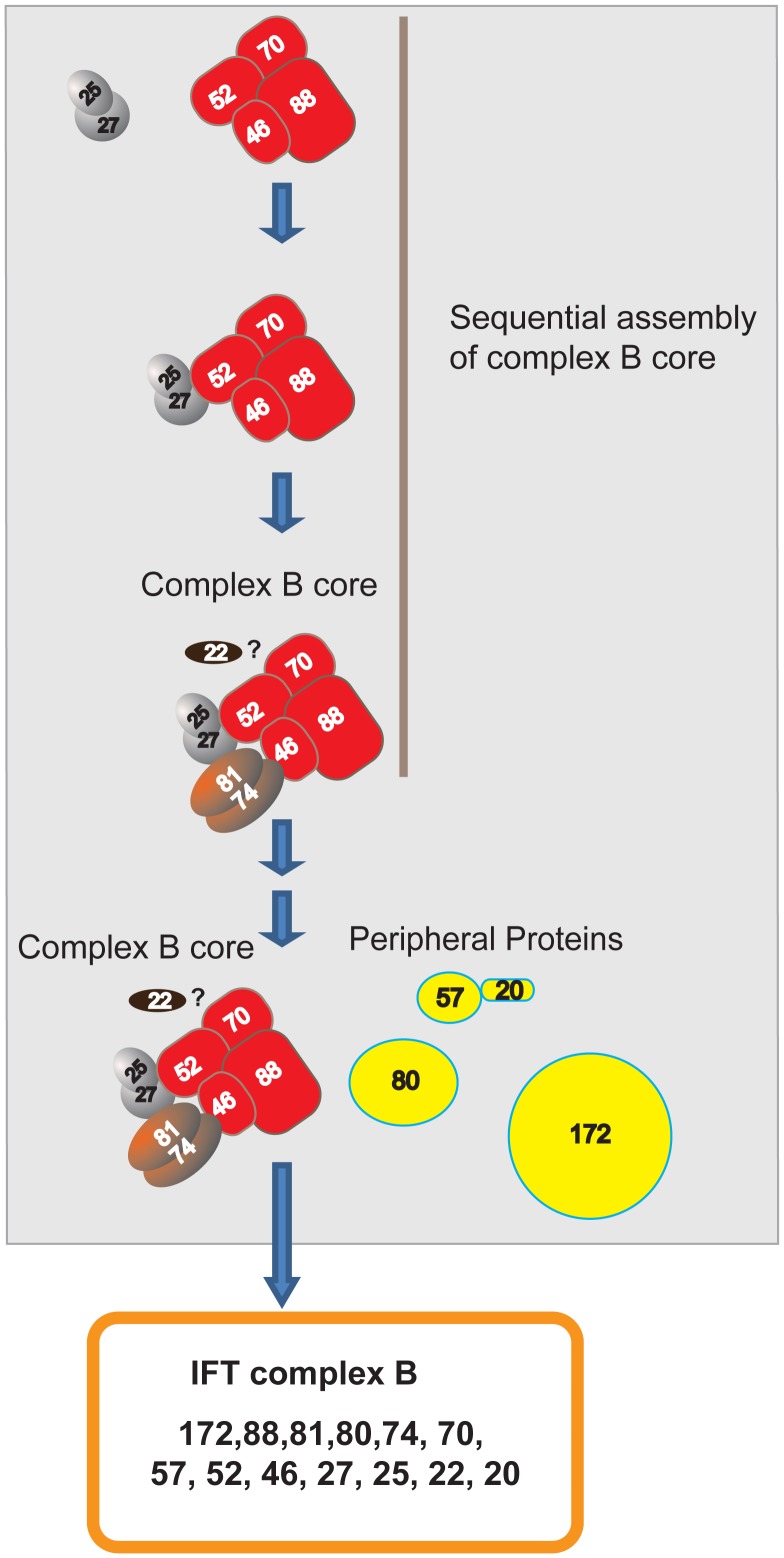
Sequential assembly and basal body localization of complex B. This model shows that during complex B assembly, complex B core assembles and then the peripheral proteins assemble onto complex B core to form an integrated complex B. First, the subcomplexes IFT88/70/52/46 and IFT27/25 binds to form IFT88/70/52/46/27/25, and the binding is dependent on IFT52. The absence of IFT52, as seen in *bld1* mutant, prevents the formation of IFT88/70/52/46/27/25, causing the accumulation of the intermediate transition complexes IFT88/70/46 and IFT27/25. Second, IFT74 and IFT81 assemble onto IFT88/70/52/46/27/25 to form the complex IFT88/70/52/46/27/25/81/74. This step is assisted by IFT46. The absence of IFT46, as seen in *ift46* mutant, either generates unstable IFT88/70/52/27/25/81/74, which is prone to degradation or dissociates the complex into IFT88/70/52/27/25 and IFT81/74. IFT81/74 is insoluble and cannot exist on its own [13]. Thus, although the interaction between IFT74 and IFT81 is strong [12], [13], the complex 81/74 only stably forms while it assembles onto the complex B core. IFT22 is a complex B core protein (data not shown) but currently it is unknown how it assembles into the complex B core. Lastly, the peripheral proteins assemble onto the complex B core to form an intact complex B. IFT88 is not required for complex B core formation, but is essential for mediating the binding of peripheral proteins to the B core. All the assembly steps involved in forming complex B core may occur at the proximal end of the basal bodies. The translocation of complex B from the proximal end of the basal body to the transition fibers is dependent on the presence of IFT88 and proper association of peripheral proteins.

In this study, we report that three complex B core proteins of *Chlamydomonas* have distinct roles in complex B assembly and stability and apical peri-basal body localization. By analyzing the intermediate complexes formed in mutants *ift46, bld1*, and *ift88* (**Fig. 1**), this report clearly demonstrates that IFT46, IFT52, and IFT88, despite being in the same subcomplex [16], do not contribute equally to the assembly of complex B. The *ift46* mutant cells are capable of assembling a relatively intact complex B, which is likely responsible for the assembly of the short flagella. An intermediate complex, likely containing IFT88/70/52/27/25, was detected in the *ift46* mutant, which may represent a key step in complex B core assembly. Although complex B does assemble in *ift46* mutant, the assembled complex without IFT46 is highly unstable and prone to degradation (**Fig. 2D**). The lack of stability could be the leading factor causing low cellular amount of complex B proteins in *ift46* mutant, which in turn is capable of assembling only stumpy flagella. In contrast, the *bld1* mutant does not contain an assembled complex B core, indicating that IFT52 plays a key role in mediating the assembly of complex B (**Fig. 1D, E**). In *ift88* mutant cells the complex B core still assembles and remains stable (**Figs. 1C** and **2C**). However, the complex B proteins no longer localize at the transition fibers, but instead are restricted to the proximal ends of basal bodies (**Fig. 4C**). The failure of anchoring complex B to the transition fibers is likely due to the absence of IFT88 itself or one of the peripheral proteins that failed to bind to the complex B. Taken together, these results reveal that the assembly of complex B is through a step-wise process and the localization of complex B involves translocation of complex B from the proximal end to the transition fibers via an IFT88 dependent mechanism (**Fig. 8**).

The accumulation of IFT particles on the transition fibers of basal bodies, where flagella assembly initiates, is obviously advantageous to flagellar assembly. This study provides some clues as to how IFT complex B localizes to the basal body. It appears that individual IFT complex B proteins are first transported to the centrioles (**Figs. 4** **,** **5** and **6**), where they assemble into a complex, and then translocate onto transition fibers. Thus, although IFT complex B normally localizes to transition fibers, where it joins with other components to form functional IFT machinery, its centriole association is likely independent of transition fibers (**Fig. 6**). This notion is consistent with previous findings that IFT complex B proteins associate with centrioles throughout mitosis [36], [38].

### IFT88 is Critical for Mediating the Attachment of Peripheral Proteins to the Complex B Core

Although IFT88 is a subunit of complex B core, it is not needed for complex B core assembly. Instead, its function is most likely to bind peripheral proteins to the core. Compared to the interactions identified within the complex B core subunits, the interaction between IFT57 and IFT20 is the only one that has been confirmed among the peripheral proteins to date [39]. Interestingly, in *ift88* mutant, the cellular level of IFT57 is greatly reduced compared to other complex B proteins [23]. It is possible that in the absence of IFT88 and IFT57, some of of the peripheral proteins are no longer able to attach to the complex B core. Future work on the identification of which peripheral protein that interacts with IFT88 and the additional interactions among peripheral proteins should provide a complete picture on the architecture and the assembly of complex B.

### IFT88 and/or Peripheral Proteins are Essential for Anchoring Complex B on the Transition Fibers

Our results indicate that IFT88 itself, or one of the complex B peripheral proteins that failed to attach to the complex B is responsible for the translocation of complex B from the proximal end of basal bodies onto the transition fibers. This idea can be tested by examining the assembly status and the localization pattern of complex B proteins in mutants of peripheral proteins. However, the set of complex B protein mutants is far from complete, with only a handful of complex B mutants currently available. The IFT complex B proteins are correctly localized to transition fibers in temperature sensitive mutant *fla11/ift172^ts^* (data not shown). Thus, it is unlikely that IFT172 is responsible for complex B transition fiber localization, although this result needs to be confirmed once a null mutant of *ift172* is available. Recently, an *ift80* mutant (likely a null mutant) was identified; like *bld1* and *ift88* mutants, this mutant is completely flagella-less with no axonemal microtubules assembled [40]. Further studies on the assembly status and the localization pattern of complex B proteins of the *ift80* mutant should provide answers to the function of IFT80 in complex B assembly and localization.

### The Anterograde Motor Fla10-kinesin-II Must be Recycled Back to the Cell Body after Each Trip to the Flagellar Tip

IFT particles undergo constant bi-directional transport along the entire length of flagella to support flagellar assembly and maintenance. This report shows that both IFT particle proteins and the anterograde motor Fla10-kinesin-II have very low turnover rate (**Figs. 2** and **3**). By analyzing the motility of GFP tagged proteins, previous studies have observed an interesting discrepancy of the movement between the IFT particle proteins and the Fla10-kinesin-II. IFT particle proteins display both the anterograde and retrograde transport. In contrast, Fla10-kinesin-II only shows the anterograde transport. No retrograde transport can be observed for Fla10-kinesin-II [41], [42], raising the question whether Fla10-kinesin-II is discarded at the flagellar tip after completing one anterograde transport and never recycled back for reuse. Here, we show that Fla10-kinesin-II is very stable (**Fig. 3**). No protein amount change of FLA10 can be detected after 16 hours of treatment with cycloheximide. Thus, normally there must be little, if not at all, new Fla10-kinesin-II synthesized in the cell body. The protein synthesis for Fla10-kinesin-II can be up-regulated when the protein turnover rate is increased, as seen in both *fla10^ts^* and *fla3-1b* mutants. Such upregulation is likely required to replenish the depleted Fla10-kinesin-II protein due to increased degradation rate. Because the turnover rate of Fla10-kinesin-II is extremely low, it has to be reused to power numerous rounds of IFT movement. Therefore, Fla10-kinesin-II must be efficiently recycled back to the cell body after each trip to the flagellar tip.

## Materials and Methods

### Strains and Culture Conditions

Strains used as controls included *Chlamydomonas reinhardtii* wild-type strain *cc125* and cell wall deficient, but otherwise wild-type strain *cw92* (CC-503). The *cw92* strain was used for the sucrose density gradients, as the cell wall deficiency made it easier to lyse the cells for protein extraction. These control strains along with mutant strains, *ift88* (CC-3943) [23], *ift46* [27], *bld1* (CC-477) [26], *fla10* null (CC-4180), and temperature-sensitive mutants *fla10^ts^* (*fla10–1* allele, CC-1919*)* and *fla3–1* (CC-4283) were obtained from the *Chlamydomonas* Center (http://www.chlamy.org). The strains were cultured on Tris-acetate-phosphate (TAP) solid plates or TAP liquid media with constant aeration in a Conviron programmed at 22°C in continuous light with constant aeration.

### Whole Cell Protein Extracts and SDS Page Electrophoresis

20 mL TAP liquid media was inoculated with a loop of cells from a fresh (within a week old) TAP solid plate culture. Liquid cultures were grown for approximately 3 days (until medium to dark green) under the above conditions. The cultures were normalized based on the lightest colored culture by adding TAP liquid media to the others until all cultures were about the same color. 1 mL of culture was spun down at 14000 RPM for 5 minutes and the supernatant was removed. The cell pellet was then resuspended in 50 µL 1X PBS. 50 µL 2X laemmli buffer was then added and the cultures were vortexed for 30 seconds The samples were then boiled for 3 minutes, and spun down for 10 minutes at 14000 RPM. The supernatant was collected and stored at −20°C for short- term or −80°C for long-term storage. Samples were used for direct electrophoresis using SDS Page gels. 5 µL of sample was added to each well, then protein amounts were adjusted based on total lane intensity detected on gels stained with Coomassie Blue using ImageLab™ Software (BioRad).

### Cycloheximide Treatment

Cells were cultured under the conditions described above. Protein was extracted using above protocol prior to treatment to be used as control samples. The remaining culture was treated with 10 µg/ml cycloheximide and again placed in the conviron under the same culture conditions as pre-treatment. Protein was then extracted at multiple time points using the same methods used when extracting control samples.

### Western Blotting

Protein was separated on a 10% SDS Page gel, then electro-transferred to a polyvinylidene difluoride membrane. Proper transfer was tested using ponceau staining. Membranes were then blocked in 5% nonfat dry milk in TBST (10 mM Tris, pH 7.5, 166 mM NaCl, plus 0.05%Tween-20) with.02% sodium azide. Membranes were then incubated in primary antibody in blocking solution for at least 1 hour (up to one day for weaker antibodies). They were then washed 4 times for 10 minutes each time in TBST. Membranes were then incubated in secondary antibody in 5% nonfat dry milk in TBST for at least one hour, followed by an additional 4 washes using TBST. Chemiluminescence with X-ray film was used to detect antibody bands.

### Antibodies

Antibodies used in this study include antibodies against α-tubulin (clone B-5–1–2, ascites fluid; Sigma); IC69 (clone 1869A; Sigma); acetylated tubulin (clone 6–11B-1, ascites fluid; sigma). IFT polypeptide antibodies include antibodies to *Chlamydomonas reinhardtii* IFT172, IFT81, IFT139 [9], IFT122 [11], IFT74 [43] IFT46 [27], IFT25 [14], IFT27 [31] and FLA10 [9].

### Sucrose Density Gradient

500 mL of cells were grown under conditions described above. Cells were then pelleted and resuspended in autolysin and shaken under light for 1 hour or until cells separated and were able to be lysed by 1% Triton. Cells were again pelleted and stored at −80°C. After thawing on ice, cells were resuspended in 10X HMDEK with DTT, followed by serial centrifugation of the supernatant at 4°C until supernatant was a clear yellow/green. High and low density sucrose solutions that contained 1X HMDEK were prepared in 12 mL centrifuge tubes. The supernatant was placed on top of the sucrose solutions, then centrifuged at 37000 RPM at 5°C for 18 hours. A No Drop Counter apparatus was used to collect 500–550 µL fractions. Enough 10X Laemmli buffer was added to each tube to make the sample 1X laemmli buffer. Samples were stored at −20°C (or −80°C for long-term storage).

Approximate S-values were derived using the sedimentation behavior of following proteins with established S-values: the prominent ∼55 kDa large subunit of RuBisCo sedimenting at ∼19 S, FLA10 with known sedimentation at about 10 S [9], and cytoplasmic radial spoke complex at 12 S [43].

### Immunofluorescent Staining

Cells were fixed in methanol and blocked with blocking buffer (5% BSA, 1% Cold Water Fish Gelatin, 10% goat serum in 1X PBS). Cells were then incubated in primary for 4 hours at room temperature or overnight at 4°C followed by washing in 0.5X PBS. Cells were incubated in secondary antibody for 1 hour at room temperature. SlowFade Antifade Reagent (Invitrogen) was used to prevent loss of signal. Cells were viewed under an Olympus IX-70 or Nikon Diaphot inverted fluorescence microscope at 100X magnification. An Image Point CCD Camera (Photometrics) was used to capture the image through PCI Software.
